# Scientific impact of studies published in temporarily available radiation oncology journals: a citation analysis

**DOI:** 10.1186/s40064-015-0885-y

**Published:** 2015-02-24

**Authors:** Carsten Nieder, Hans Geinitz, Nicolaus H Andratschke, Anca L Grosu

**Affiliations:** Department of Oncology and Palliative Medicine, Nordland Hospital, 8092 Bodø, Norway; Institute of Clinical Medicine, Faculty of Health Sciences, University of Tromsø, 9038 Tromsø, Norway; Department of Radiation Oncology, Krankenhaus der barmherzigen Schwestern and Medical Faculty, Johannes Kepler University Linz, 4010 Linz, Austria; Department of Radiation Oncology, University Hospital Zurich, 8091 Zurich, Switzerland; Department of Radiation Oncology, University Hospital Freiburg, 79106 Freiburg, Germany

**Keywords:** Radiation oncology, Radiotherapy, Research evaluation, Scientific publishing, Citation

## Abstract

The purpose of this study was to review all articles published in two temporarily available radiation oncology journals (Radiation Oncology Investigations, Journal of Radiosurgery) in order to evaluate their scientific impact. From several potential measures of impact and relevance of research, we selected article citation rate because landmark or practice-changing research is likely to be cited frequently. The citation database Scopus was used to analyse number of citations. During the time period 1996-1999 the journal Radiation Oncology Investigations published 205 articles, which achieved a median number of 6 citations (range 0-116). However, the most frequently cited article in the first 4 volumes achieved only 23 citations. The Journal of Radiosurgery published only 31 articles, all in the year 1999, which achieved a median number of 1 citation (range 0-11). No prospective randomized studies or phase I-II collaborative group trials were published in these journals. Apparently, the Journal of Radiosurgery acquired relatively few manuscripts that were interesting and important enough to impact clinical practice. Radiation Oncology Investigations’ citation pattern was better and closer related to that reported in several previous studies focusing on the field of radiation oncology. The vast majority of articles published in temporarily available radiation oncology journals had limited clinical impact and achieved few citations. Highly influential research was unlikely to be submitted during the initial phase of establishing new radiation oncology journals.

## Background

Scientifc publishing is an important task for radiation oncologists pursuing an academic career (Holliday et al. 2014). Many of these physicians or physician-scientists undergo yearly rating, and are more or less forced to produce a certain number of publications or surrogate achievements such as impact factor (Holliday et al. 2013). Collaborative groups competing for research funding also feel the pressure of succeeding with clinical trials and publishing their results in prestigious journals (Nieder 2012). Many well-established journals with high impact factor have low acceptance rates of submitted manuscripts. Continuous increases in number of submissions and competition for space result in a need for additional publication channels. Publishing companies launching new journals face the challenges of attracting a sufficient number of scientifically sound manuscripts, achieving high visibility in search engines, and achieving indexing in databases such as PubMed. A rapidly increasing impact factor is also helpful for the continued success of new journals. During the second half of the 1990s, when the field was dominated by the “International Journal of Radiation Oncology, Biology and Physics” and “Radiotherapy and Oncology”, two attempts were made to establish additional specialty journals, “Radiation Oncology Investigations” and “Journal of Radiosurgery”. However, these traditional print journals without page charges did not succeed and were taken off the market after few volumes. We hypothesized that failure was caused by insufficient number of publications that were interesting and important enough to attract readers and impact clinical practice, and that this lack of appeal would be reflected by low number of citations for most published studies. In order to evaluate this hypothesis, patterns of citation for these two journals were analysed.

## Materials and methods

A systematic search of the citation database Scopus (Elsevier B.V., http://www.elsevier.com/online-tools/scopus) by use of the function ‘document search’ was performed on 3^rd^ of July 2014. All articles published in “Radiation Oncology Investigations” and the “Journal of Radiosurgery” were selected irrespective of subject area, document and article type (review, clinical study, experimental study, case report etc.). Then, these articles were ranked by number of citations (field ‘times cited’ in the Scopus citation database). As requested during manuscript review, we also performed comparisons to the “International Journal of Radiation Oncology, Biology and Physics”. The respective Scopus search was performed on 6^th^ of February 2015.

## Results

**Figure 1:**
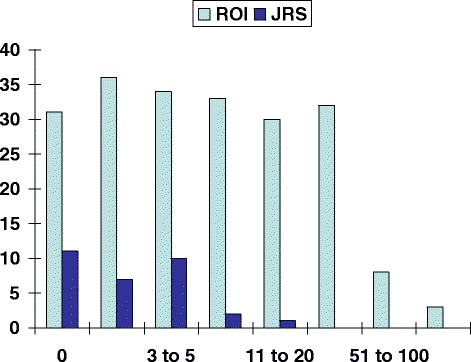
**Patterns of citation of all articles published in Radiation Oncology Investigations (ROI) and the Journal of Radiosurgery (JRS).**

During the time period 1996-1999 “Radiation Oncology Investigations” published 205 articles, which achieved a median number of 6 citations (range 0-116). Figure 1 shows the distribution of citations. References (Seymour & Mothersill 1997; Sheridan et al. 1997; Desai et al. 1998; Schmidt-Ullrich et al. 1999; Smith & Haffty 1999; Johnson et al. 1998; Monga et al. 1999; Stickle et al. 1999; Merrick et al. 1998; Roach et al. 1997; Wazer et al. 1999; Joschko et al. 1997; Chidel et al. 1999; Durand & Olive 1997; Peschel et al. 1999; Norman et al. 1997; Kramer et al. 1998; Leborgne et al. 1997; Epperly et al. 1999; Chancy et al. 1998; Prete et al. 1998; Fernandez-Vicioso et al. 1997; Kang & Suh 1999; Banasiak et al. 1999; Nathu et al. 1998; Haffty et al. 1997; Gieger et al. 1997) and Table 1 include the 25 most frequently cited articles (all had at least 30 citations and were published in volumes 5-7, most emanated from the USA). The most frequently cited article in the first 4 volumes achieved 23 citations (Teicher et al. 1996). As also shown in Table 1, the median number of authors was 5 (range 2-10). Both clinical, dosimetric and radiobiological research as well as reviews achieved at least 30 citations. The most common topics were radiobiology (n = 8) and prostate cancer (n = 6). Topics covered all technological developments of that decade, e.g. brachytherapy, stereotactic radiotherapy and intensity-modulated radiotherapy. No final results of randomized studies or phase I-II collaborative group trials were published. The most common type of research were retrospective clinical studies (n = 10, Table 1). The median number of patients in these studies was limited (40.5, range 3-123).

**Table 1:** **The most frequently cited articles published in “Radiation Oncology Investigations” (in order of citation frequency, references 4-30)**

**Short title**	**Authors (number of authors)**	**Country of origin**	**Topic**	**Design**	**Comments**
Mutations and genomic instability	Seymour CB and Mothersill C (2)	Ireland	Radiobiology	In vitro study	
Radiosensitivity in squamous cell cancer	Sheridan MT et al. (4)	Ireland	Radiobiology	In vitro study	
Morbidity following prostate brachytherapy	Desai J et al. (5)	USA	Prostate cancer	Retrospective clinical study	117 patients
Accelerated repopulation	Schmidt-Ullrich RK et al. (8)	USA	Radiobiology	Review	
Molecular markers in head and neck cancer	Smith BD and Haffty BG (2)	USA	Head and neck cancer	Review	Review of prognostic factors
Bulky cervical lymphadenopathy	Johnson CR et al. (4)	USA	Head and neck cancer	Retrospective clinical study	81 patients
Fatigue in patients with prostate cancer	Monga U et al. (4)	USA	Prostate cancer	Prospective clinical study	36 patients, external beam irradiation
Prevention of esophagitis	Stickle RL et al. (5)	USA	Radiobiology	Mouse model	plasmid/liposome delivery of the human manganese superoxide dismutase transgene
Prostate brachytherapy	Merrick GS et al. (4)	USA	Prostate cancer	Dosimetric evaluation	10 patients
Prostate volumes and movement	Roach M 3^rd^ et al. (3)	USA	Prostate cancer	Dosimetric evaluation	10 patients
Positive margins and local recurrence	Wazer DE et al. (6)	USA	Breast cancer	Retrospective clinical study	105 patients
Radiation plus gemcitabine	Joschko MA et al. (7)	Australia	Radiobiology	Mouse model	Squamous cell carcinoma
Single brain metastases	Chidel MA et al. (5)	USA	Lung cancer	Retrospective clinical study	33 patients
Tirapazamine	Durand RE and Olive PL (2)	Canada	Radiobiology	Mouse model	Physiologic and cytotoxic effects
Prostate brachytherapy	Peschel RE et al.(4)	USA	Prostate cancer	Retrospective clinical study	123 patients
Canine brain tumors	Norman A et al. (6)	USA	Brain tumors	Retrospective study	Irradiation in pet dogs
Radiosurgery vs. intensity-modulated RT	Kramer BA et al. (5)	USA	Brain tumors	Dosimetric evaluation	Irregularly shaped targets
Biologically effective doses	Leborgne F et al. (5)	Uruguay	Cervical cancer	Radiobiology data	Evaluation of clinical brachytherapy data
Overexpression of MnSOD	Epperly MW et al. (6)	USA	Radiobiology	In vitro study	Hematopoietic progenitor cells
Phyllodes tumor of breast	Chancy AW et al. (4)	USA	Phyllodes tumor	Retrospective clinical study	8 patients
Prostate brachytherapy	Prete JJ et al. (5)	USA	Prostate cancer	Dosimetric evaluation	15 patients
Radiosurgery for single brain metastases	Fernandez-Vicioso E et al. (5)	USA	Brain tumors	Retrospective clinical study	48 patients
Neurosarcoidosis	Kang S and Suh JH (2)	USA	Neurosarcoidosis	Retrospective clinical study	3 patients
Cellular radiosensitivity	Banasiak D et al.(5)	Australia	Radiobiology	In vitro study	Bladder cancer and ureteral cells
Merkel cell carcinoma	Nathu RM et al. (3)	USA	Skin cancer	Retrospective clinical study	24 patients
Porfiromycin	Haffty BG et al. (10)	USA	Head and neck cancer	Phase I and III data	Acute toxicity interim analysis of a phase III study
Radiosurgery of melanoma metastases	Gieger M et al. (6)	USA	Brain tumors	Retrospective clinical study	12 patients

The “Journal of Radiosurgery” published 31 articles, all in the year 1999, which achieved a median number of 1 citation (range 0-11, Figure 1). References (Garell et al. 1999; Maire et al. 1999; Gibon et al. 1999; Solberg et al. 1999; Sanghavi et al. 1999) include the 5 most frequently cited articles (all had at least 5 citations and were published in volume 2). Both clinical and physics research as well as reviews achieved any citations. No randomized studies or phase I-II collaborative group trials were published.

The “International Journal of Radiation Oncology, Biology and Physics” published almost 2200 articles during the time period 1996-1999. These achieved a median number of 22 citations (range 0-1135). Overall, 9% of articles achieved at least 100 citations (p = 0.0002 in Chi-Square test comparing the three journals).

## Discussion

The objective of this review was to identify pattern of scientific publication in two radiation oncology journals which were only temporarily available. While Radiation Oncology Investigations covered the whole field of radiation oncology, the Journal of Radiosurgery had a much narrower focus. After arbitrary decisions about which database to search (only those providing citation numbers could be considered for the purpose of this review), we performed a comprehensive evaluation of published research and number of citations accumulated during more than a decade after publication of the final journal volume. These citation numbers can be considered mature because previous analyses demonstrated that citation rate is gradually increasing for the first years after publication, followed by rapid decline (except for landmark randomized studies, which change clinical practice) (Stringer et al. 2010; Kondziolka 2011). Citation rate of published articles was chosen to define the most important contributions. Articles with high numbers of citations are likely those that impressed other clinicians/scientists and had impact on clinical practice or future developments in the field (Shao et al. 2013). It should be noticed that searches in different databases will result in more or less variable citation counts and that the present results therefore provide only a snapshot. This has been illustrated, e.g., in a cohort study of 328 articles published in JAMA, Lancet, or the New England Journal of Medicine between October 1, 1999, and March 31, 2000 (Kulkarni et al. 2009). Total citation counts for each article up to June 2008 were retrieved from Web of Science, Scopus, and Google Scholar. Google Scholar and Scopus retrieved more citations per article with a median of 160 and 149, respectively, than Web of Science (median 122, p < 0.001 for both comparisons). Importantly, Web of Science, Scopus and Google Scholar produced quantitatively and qualitatively different citation counts.

In a previous study of radiosurgery for various conditions, 1.5% of all articles (time period 1951-2010) achieved more than 100 citations (Kondziolka 2011). This figure corresponds well to that of articles in Radiation Oncology Investigations (3 out of 205, 1.5%), while the “International Journal of Radiation Oncology, Biology and Physics” achieved significantly higher proportions during the same time interval (1996-1999; 9%). The Top 100 radiation oncology articles from the time period 1999-2001 achieved a median of 208 citations (range 121-1149) (Nieder et al. 2013a), i.e. more than any of the publications reviewed here. When comparing articles published during different time periods, sources of bias must be acknowledged (variable temporal patterns of citation, increasing use of online databases in recent years, better access to published articles).

An analysis restricted to German radiation oncology publications revealed that most citations per year since publication were recorded for meta-analyses and randomized phase III trials (Nieder 2012). Lower figures were recorded for review articles, non-phase III prospective clinical trials, and retrospective clinical studies. Another analysis demonstrated that pattern of publication of the most influential radiation oncology studies was dominated by only two scientific journals: the Journal of Clinical Oncology and the International Journal of Radiation Oncology Biology and Physics (Nieder et al. 2013a). Several newly launched journals (first issue after 1999) managed to attract highly cited articles (Lancet Oncology, Nature Reviews Clinical Oncology or Nature Reviews Cancer, which had rapidly increasing impact factors). Apparently, despite controversy around impact factors and optimal evaluation of research productivity and quality (Kanaan et al. 2011; Durieux & Gevenois 2010), researchers find it attractive and desirable to publish their most important radiation oncology related work in the top journals of the field. The present findings are in line with these considerations.

Highly cited work is unlikely to be published in the first volumes of new journals. Prospective randomized trials and non-randomized trials performed by influential collaborative groups are prefentially submitted to well-established journals. It has also been shown that five newly established oncology journals, in this case open access publications without print issues, all published less than 50 articles in their first annual volume (Nieder et al. 2013b). First after 4-5 years number of articles per volume increased sharply. At that time, the two journals analysed here were already taken off the market. During the time period in question (1996-1999), no other new radiation oncology journals entered the market. However, afterwards two successful journals were established (in 2006 “Radiation Oncology” (open access, no print issues); in 2011 “Practical Radiation Oncology” (print issues, sister journal to the “International Journal of Radiation Oncology, Biology and Physics”)). Regarding “Radiation Oncology”, the annual volumes 2006-2008 all consisted of less than 50 articles. In 2009, contents expanded to 71 articles, while the most recent volume (2014) featured approximately 300. On the one hand, one could hypothesize that open access fees might prevent authors from submitting to journals like “Radiation Oncology”. On the other hand, advantages such as rapid publication and unlimited distribution and access appear to outweigh financial considerations. Institutional membership or publication funds and waiver of fees for authors from countries with limited resources might also encourage researchers to publish in open access journals.

## Conclusions

Highly cited research typically appears in relatively few well-established journals. The vast majority of articles published in temporarily available radiation oncology journals had limited clinical impact and achieved few citations, especially those accepted in the initial phase of marketing.
